# Classification of the Poor in Workhouses

**Published:** 1889-11-09

**Authors:** 


### The Hospital

A Weekly Institutional Journal of

**Science, Medicine, Nursing, and Philanthropy.**

*For Hospitals, Asylums, Medical Practitioners, Students, Nurses, Families, Parishes, Congregations, and the Charitable Public.*

Vol. VII. No. 163.] [November 9, 1889.

### CLASSIFICATION OF THE POOR IN WORKHOUSES.

It has long been England's boast that no man or woman need starve in this country except by their own choice. The Poor Law system was specially instituted to secure this result at least, and whatever its defects—and they are many—the boast may be taken as literally, though not accurately, true in substance and in fact. Indeed, when Louise Michel came to London a few years ago to preach her propaganda of socialism and revolution, she is stated to have given up her crusade in despair on the ground that owing to the Poor Law the people were not miserable enough to make her doctrines acceptable, or even applicable to the mass of the poor. Be this as it may, it must not be forgotten that there are 30 to 50 people die every year in London alone through starvation. Why is this ? Mainly because the respectable poor, who through sickness or other causes not due to vice or idleness are reduced to want, will not enter the workhouse through their repugnance to associate with the depravity of those usually to be found there. It is an undoubted fact that the persons whom the Poor Law ought to help first—the really deserving poor—are very difficult to get at. The guardians do, indeed, deal with a vast amount of destitution produced by idleness, drunkenness, and vice, but they fail to reach, as Mr. M'Dougall, jun., of Manchester, recently pointed out, a large proportion of the unfortunate poor who are reduced to want through causes which do not arise from any personal fault. The dread of vicious companionship is at present the real workhouse test, and we must never fail to remember that great numbers of people in large towns live on the verge of absolute want, and are, in fact, a great deal worse off than the inmates of the union. This is so, because it is a cardinal rule in the administration of poor relief that none but those who are really forced upon the guardians are dealt with at all. The Poor Law officers are not expected to go out of their way to meet destitution or poverty, although, as Mr. Jacson, the vice-chairman of the Lancashire County Council recently pointed out, many of those who pay rates are themselves little above the rank of pauperism. Such are the facts, and it may be well to consider if a remedy can be found for the existing evils of the Poor Law administration.

The Poor Law system at present fails in the workhouses, because people of all classes and grades are mixed together without any regard to moral, social, physical, or other classification, though this is to be found in every other class of institution throughout the country. No one would dream of attempting to educate children without grouping them according to their respective abilities and intelligence. It must surely be wrong, therefore, to treat adults dependent upon the Poor Law without regard to similar consideration, if the aim of the system be to build up and reclaim, not to degrade and keep under. It is scarcely credible, however, that not only is there no classification in workhouses, but that in the Poor Law infirmaries—which structurally are almost perfect—the administration is equally faulty. Instances have been brought under our notice where patients belonging to the respectable classes have unfortunately found themselves in a ward of a Poor Law infirmary within the metropolis. In one instance—that of a young girl, a domestic servant —we remember the effects of association with the immoral and depraved in the infirmary ward were so terrifying, that she left the building at the risk of her life rather than submit to them. This state of things is the more surprising, because, in regard to nursing and treatment, as well as to structural arrangements, the best of the Poor Law infirmaries leave little, if anything, to be desired. If you enter a ward in the daytime, everything seems to be in perfect order and the patients look as comfortable as possible. It is only when the nurses and officers are out of the way that the atmosphere of the ward sinks to the level of the most depraved, who are usually the most overbearing, of its inmates. Here, again, classification on intelligent lines would prevent a gross injustice being perpetrated upon many poor people which at present leads to their temporary defilement, if not to their permanent injury.

Again, it must not be forgotten that the workhouse, and we fear it may be possible the Poor Law infirmary also, is the recruiting ground for the vicious. We have been assured by some of the most intelligent officers connected with metropolitan workhouses that recruiting sergeants of both sexes are sent into the workhouse by the keepers of improper houses, by representatives of thieves, and other depraved agencies, for the purpose of picking up likely recruits to aid them in their vile trades and occupations. People have but little idea of the amount of energy and persistence with which the organisers of vice pursue their trade of recruiting from the young, inexperienced, and unprotected of both sexes in all directions throughout a city like London. We have been assured, for instance, that there are to be found in the neighbourhood of the docks at the present time representatives of vicious houses disguised as Sisters of Mercy who board vessels bringing emigrants, and by their arts and dress deceive many, who are thus lured to destruction. An agency conducted under the supervision of some of the most noble of Jewish women is labouring hard to look after the unprotected who arrive in the docks, but their work has been interfered with, we understand, by the practices of the unworthy creatures who pass themselves off as Sisters of Mercy. This is a matter which demands the early and searching investigation of Cardinal Manning and those who are responsible for religious sisterhoods in this country to-day.

Things being as we have here represented them, it may well be asked if any remedy can be found and applied, so as to make the Poor Law system better able to meet the case of the really deserving poor, who are temporarily disabled from earning a living, by causes altogether beyond their own control. There can be no doubt that an intelligent board of guardians may do much to meet this evil in the district under its immediate charge. The Paddington Guardians, for example, have summoned a conference of the ratepayers and others interested in the poor, with the view of seeing how far the administration of relief in their district may be made to better meet the requirements of the poorer population. They hope to be able to organise a better system for dealing with applications for relief, by drawing a sharp distinction between the deserving and the undeserving. They believe that the workhouse should alone be offered to the latter, whilst out-relief, upon certain conditions, may be properly granted to the former. They further wish to act in co-operation with the almoners of charities throughout the parish of Paddington, with the view of assisting in the best possible way all deserving cases which may arise from time to time. The conference considered what is the best way to deal with (1) the aged and infirm deserving poor, and (2) with deserving widows with families. We are grateful to the Guardians of Paddington for the step they have taken, and believe that it will lead to important results. Speaking generally, however, there can be no question that arrangements must be made to classify the poor in workhouses—that is to say, there must be a classification of character as well as one of bodily condition. It is monstrous that any respectable poor person forced to enter a workhouse should be compelled to associate with the vicious, and it is a hopeful sign, that the majority of the poorer population prefer to face great misery outside, rather than suffer contamination by the associations to be found within the workhouse itself.

Of course, the difficulty the guardians have to meet is due to the fact that workhouses, as at present constructed, are not adapted to classification of the kind proposed. Further, the ratepayers would, not unnaturally, object to any further large expenditure upon buildings for Poor Law purposes, seeing that those already in existence are usually more than adequate to meet the requirements of the union in which they are situated. We must therefore temporise a little, until the County Councils have got into working order, when it should be possible to hand over to them the whole of the Poor Law organisation within the districts they control. If this were done, then the workhouses could be divided, and an accurate classification—without any further cost, and according to character as well as condition—could easily be made and sustained. The advantages of this arrangement will be apparent to those who are familiar with the administration of the Poor Law in the metropolis. The benefits would be equally great, however, in the counties, because a county—like Lancashire, for instance—which has so many workhouses scattered up and down throughout its area, and varying in size from 20 to 300 and even 1,000 beds, that it would not be difficult to arrange for a very complete system of classification without any increased cost to the ratepayers. There can be no doubt that a thorough system of classification in regard to workhouses will have to be established and enforced in the near future. For our own part we feel it is far from creditable to the present generation that the difficulties surrounding this important question have not been overcome long ago.

